# Supplementary material for: Natural language processing models that automate programming will transform chemistry research and teaching
Source: Digit Discov. 2022 Feb 3;1(2):79–83. doi: 10.1039/d1dd00009h (PMC8996826; doi:10.1039/d1dd00009h)
Supplement: DD-001-D1DD00009H-s001 [file DD-001-D1DD00009H-s001.pdf]

Electronic Supplementary Material (ESI) for Digital Discovery.
This journal is © The Royal Society of Chemistry 2022

Supporting Information for Natural Language Processing Models That Automate Programming Will Transform Chemistry Research and Teaching

Glen M. Hocky
Department of Chemistry, New York University

Andrew D. White
Department of Chemical Engineering, University of Rochester

Unless otherwise specified, the `davinci-codex` model was used with $T = 0$. Codex and other LLMs output a probability distribution, so that multiple outcomes can be generated using top-k algorithms that select the k most likely outcomes. This is how we have generated multiple outcomes from a given prompt. Temperature also plays a role in which outcomes are generated, with higher temperatures flattening the probability distribution and enabling less likely samples.

Dissociation energy

Here, $T = 0.1$ for each case. The first two codes appear to run correctly and give full dissociation curves. The third fails in setting up the optimizer due to a "Trust radius got too small" error, so it would need some editing to work properly. Result 2 also illustrates the kind of subtle scientific errors which could arise. In this example, the reference energy used is the nuclear repulsion energy. While this does not change the results in this case, it would be more correct/standard to make a plot with the reference energy as the fully dissociated state.

Result 1 (used to make Fig. 1):

```
"""
Compute the dissociation curve of H2 using the pyscf library
"""
import numpy as np
import matplotlib.pyplot as plt
from pyscf import gto, scf

# Define the molecule
mol = gto.M(atom='H 0 0 0; H 0 0 1.4', basis='sto-3g')

# Perform the SCF calculation
scf_eng = scf.RHF(mol)
scf_eng.conv_tol = 1e-9
scf_eng.conv_tol_grad = 1e-7
scf_eng.kernel()

# Plot the dissociation curve
dissociation_curve = np.arange(0.5, 3.0, 0.1)
energies = []
for r in dissociation_curve:
    mol = gto.M(atom='H 0 0 0; H 0 0 '.format(r), basis='sto-3g')
    scf_eng = scf.RHF(mol)
    scf_eng.conv_tol = 1e-9
    scf_eng.conv_tol_grad = 1e-7
    scf_eng.kernel()
    energies.append(scf_eng.e_tot)

plt.plot(dissociation_curve, energies)
plt.xlabel('Length of bond (Angstrom)')
plt.ylabel('Total energy (Hartree)')

plt.show()
```

Result 2

```
"""
Compute the dissociation curve of H2 using the pyscf library
"""
import numpy as np
import pyscf
from pyscf import gto, scf, dft

# Define the molecule
mol = gto.M(atom='H 0 0 0; H 0 0 1.4', basis='sto-3g', verbose=3)

# Compute the reference energy
en_ref = mol.energy_nuc()

# Compute the dissociation curve
r = np.linspace(0.5, 2.0, 100)
en = []
for b in r:
    mol.atom = [['H', (0, 0, 0)], ['H', (0, 0, b)]]
    mol.build()
    mf = scf.RHF(mol)
    mf.kernel()
    en.append(mf.e_tot - en_ref)

# Plot the dissociation curve
import matplotlib.pyplot as plt
plt.plot(r, en)
plt.xlabel('Bond length (Bohr)')
plt.ylabel('Energy (Hartree)')
plt.show()
```

Result 3:

```
"""
Compute the dissociation curve of H2 using the pyscf library
"""

import numpy as np
import pyscf
from pyscf import gto, scf, dft, cc, mp
from pyscf.geomopt.berny_solver import optimize

mol = gto.Mole()
mol.atom = """
H 0.0 0.0 0.0
H 0.0 0.0 0.75
"""
mol.basis = "6-31G"
mol.verbose = 0
mol.build()

mf = scf.RHF(mol)
mf.kernel()

# Optimize the geometry of the molecule
mol_eq = optimize(mp.MP2(mf))

# Compute the dissociation curve
r_array = np.arange(0.6, 4.0, 0.1)
e_array = []
for r in r_array:
    mol_eq.atom = [['H', (0, 0, 0)], ['H', (0, 0, r)]]
```

```
mol_eq.build()
e_array.append(mol_eq.kernel())
```

Caffeine from SMILES

Result 1: Used to make Fig. 3(A)

```
"""
1. Load the caffeine molecule from SMILES
2. Draw it
"""
from rdkit import Chem
from rdkit.Chem import Draw
from rdkit.Chem.Draw import IPythonConsole

IPythonConsole.ipython_useSVG = True

caffeine = Chem.MolFromSmiles('CN1C=NC2=C1C(=O)N(C(=O)N2C)C')

Draw.MolToImage(caffeine)
```

Result 2:

```
"""
1. Load the caffeine molecule from SMILES
2. Draw it
"""
from rdkit import Chem
from rdkit.Chem import Draw
from rdkit.Chem.Draw import IPythonConsole
from rdkit.Chem import Descriptors

# Load the caffeine molecule from SMILES
mol = Chem.MolFromSmiles('CN1C=NC2=C1C(=O)N(C(=O)N2C)C')

# Draw it
Draw.MolToImage(mol)
```

2D random walk

Full example from Fig. 3(C). The first part produces the image on the left. The second follow up request to write a function produces the figure on the right, followed by other trials that don't necessarily do exactly what the generated comments say.

```
"""
This script generates the behavior of a 2D random walk and plots trajectories
"""
import numpy as np
import matplotlib.pyplot as plt

# Parameters
N = 1000 # number of steps
x = np.zeros(N) # x position
y = np.zeros(N) # y position

# Random walk
for i in range(1, N):
    r = np.random.rand()
    if r < 0.25:
        x[i] = x[i-1] + 1
        y[i] = y[i-1]
    elif r < 0.5:
        x[i] = x[i-1] - 1
        y[i] = y[i-1]
    elif r < 0.75:
        x[i] = x[i-1]
        y[i] = y[i-1] + 1
    else:
        x[i] = x[i-1]
        y[i] = y[i-1] - 1

# Plot
plt.plot(x, y)
plt.show()

"""
Rewrite this into a function
"""
def random_walk(N):
    x = np.zeros(N)
    y = np.zeros(N)
    for i in range(1, N):
        r = np.random.rand()
        if r < 0.25:
            x[i] = x[i-1] + 1
            y[i] = y[i-1]
        elif r < 0.5:
            x[i] = x[i-1] - 1
            y[i] = y[i-1]
        elif r < 0.75:
            x[i] = x[i-1]
            y[i] = y[i-1] + 1
        else:
            x[i] = x[i-1]
            y[i] = y[i-1] - 1
    return x, y

# Plot
x, y = random_walk(N)
plt.plot(x, y)
plt.show()

# Plot multiple trajectories
for i in range(10):
    x, y = random_walk(N)
    plt.plot(x, y)
plt.show()

# Plot multiple trajectories with different colors
for i in range(10):
    x, y = random_walk(N)
    plt.plot(x, y, color='black')
plt.show()

# Plot multiple trajectories with different colors and markers
for i in range(10):
    x, y = random_walk(N)
    plt.plot(x, y, color='black', marker='o')
plt.show()
```

Chemical entity recognition example from Fig 2

GPT-3 was used with T=0.7

```
This code extracts chemical entities:
Sentence:  Negatively charged PS particles were produced in the same fashion, replacing
2,2'-azobis(2-methylpropionamidine) dihydrochloride with an equivalent weight amount of
potassium persulfate (≥99% from MilliporeSigma) Chemical Entities:
2,2'-azobis(2-methylpropionamidine) dihydrochloride , potassium persulfate
---
Sentence:  Degassed CHCl3 (250 mL) was added via canula and the solution was brought to
reflux for 10 hours.
Chemical Entities:  CHCl3
---
Sentence:  The solution was washed with 1 M NaOH and dried with MgSO4.
Chemical Entities:  NaOH, MgSO4
---
Sentence:  In brief, fluorescent dyes dissolved in tetrahydrofuran (THF) were added to
particles stabilized with Pluronic F108 (MilliporeSigma) to a final concentration of 30% v/v
THF, then diluted by a factor of five before washing the particles via multiple sedimentation
and resuspension cycles to set them in pure water.
Chemical Entities:  Pluronic F108, tetrahydrofuran, THF
```

Electronic structure input file generation

Multiple trials at generating input files with Gaussian, and one without Gaussian specified. All three require importing Chem from rdkit and AllChem from rdkit.Chem for the python code to operate, and the third requires importing subprocess. The first example runs and produces an input file which can be executed by gaussian. The second does not, atom objects do not have functions such as GetX. The third is able to produce an xyz file properly, but does not make an input file for psi4, and hence fails on the subprocess line.

```
def gauss(smiles):
    """
    This function prepares a Gaussian input file from the given smiles using rdkit to prepare initial coordintes by
    1. Converting SMILES to molecule
    2. Preparing 3D coordinates
    3. Writing out Gaussian input file
    """
    # Convert SMILES to molecule
    mol = Chem.MolFromSmiles(smiles)
    # Generate 3D coordinates
    mol = Chem.AddHs(mol)
    AllChem.EmbedMolecule(mol)
    AllChem.MMFFOptimizeMolecule(mol)
    # Write out Gaussian input file
    file_name = smiles + '.com'
    with open(file_name, 'w') as f:
        f.write('%chk=' + smiles + '.chk\n')
        f.write('%nprocshared=4\n')
        f.write('%mem=4GB\n')
        f.write('#p opt b3lyp/6-31g(d) empiricaldispersion=gd3\n\n')
        f.write(smiles + '\n\n')
        f.write('0 1\n')
        for atom in mol.GetAtoms():
            pos = mol.GetConformer().GetAtomPosition(atom.GetIdx())
            f.write(':<4 :>10.5f :>10.5f :>10.5f\n'.format(atom.GetSymbol(), pos.x, pos.y, pos.z))
        f.write('\n\n')
    return file_name
```

```
def gauss(smiles):
    """
    This function prepares a Gaussian input file from the given smiles using rdkit to prepare initial coordintes by
    1. Converting SMILES to molecule
    2. Preparing 3D coordinates
    3. Writing out Gaussian input file
    """
    # Convert SMILES to molecule
    mol = Chem.MolFromSmiles(smiles)
    # Generate 3D coordinates
    mol = Chem.AddHs(mol)
    AllChem.EmbedMolecule(mol, useExpTorsionAnglePrefs=True, useBasicKnowledge=True)
    # Write out Gaussian input file
    file_name = smiles + '.com'
    file = open(file_name, 'w')
    file.write('%mem=5gb\n')
    file.write('%nproc=8\n')
    file.write('#p opt b3lyp/6-31g(d) empiricaldispersion=gd3\n')
    file.write('\n')
    file.write('Title Card Required\n')
    file.write('\n')
    file.write('0 1\n')
    for atom in mol.GetAtoms():
        file.write(str(atom.GetAtomicNum()) + ' ' + str(atom.GetX()) + ' ' + \
            str(atom.GetY()) + ' ' + str(atom.GetZ()) + '\n')
    file.write('\n')
    file.close()
    return file_name

def run_gauss(file_name):
    """
    This function runs Gaussian on the given input file and returns the energy
```

```
    """
    # Run Gaussian
    os.system('g09 < ' + file_name + ' > ' + file_name + '.log')
    # Read energy from Gaussian output file
    file = open(file_name + '.log', 'r')
    lines = file.readlines()
    file.close()
    for line in lines:
        if 'EUMP2 =' in line:
            energy = float(line.split()[2])
    return energy

def get_energy(smiles):
    """
    This function returns the energy of the given molecule
    """
    file_name = gauss(smiles)
    energy = run_gauss(file_name)
    return energy
```

```
def single_point(smiles):
    """
    This function prepares a quantum chemistry input file from the given smiles using rdkit to prepare initial coordintes
    """
    # Generate the 3D structure of the molecule
    mol = Chem.AddHs(Chem.MolFromSmiles(smiles))
    AllChem.EmbedMolecule(mol)
    AllChem.MMFFOptimizeMolecule(mol)

    # Write the coordinates to a file
    with open('coords.xyz', 'w') as f:
        f.write(str(mol.GetNumAtoms()) + '\n\n')
        for atom in mol.GetAtoms():
            pos = mol.GetConformer().GetAtomPosition(atom.GetIdx())
            f.write(' :10.4f :10.4f :10.4f\n'.format(atom.GetSymbol(), pos.x, pos.y, pos.z))

    # Run the quantum chemistry calculation
    subprocess.call(['psi4', 'coords.xyz', '-o', 'output.dat'])

    # Read the output file
    with open('output.dat', 'r') as f:
        lines = f.readlines()

    # Get the energy
    energy = float(lines[-1].split()[1])

    # Get the coordinates
    coords = []
    for line in lines[-(mol.GetNumAtoms() + 2):-2]:
        words = line.split()
        coords.append([float(words[1]), float(words[2]), float(words[3])])
    return energy, coords

def get_energy(smiles):
    """
    This function returns the energy of the molecule in kcal/mol
    """
    energy, _ = single_point(smiles)
    return energy * 627.509
```
